# Fever in the ICU: A Predictor of Mortality in Mechanically Ventilated COVID-19 Patients

**DOI:** 10.1177/0885066620979622

**Published:** 2020-12-15

**Authors:** Rachel L. Choron, Christopher A. Butts, Christopher Bargoud, Nicole J. Krumrei, Amanda L. Teichman, Mary E. Schroeder, Michelle T. Bover Manderski, Jenny Cai, Cherry Song, Michael B. Rodricks, Matthew Lissauer, Rajan Gupta

**Affiliations:** 1 Division of Acute Care Surgery, Department of Surgery, 43982 Rutgers Robert Wood Johnson Medical School, New Brunswick, NJ, USA; 2 Division of Acute Care Surgery, Froedtert Memorial Lutheran Hospital, Medical College of Wisconsin, Milwaukee, WI, USA; 3 Department of Biostatistics and Epidemiology, Rutgers School of Public Health, Piscataway, NJ, USA

**Keywords:** COVID-19, coronavirus, ICU, critically ill, fever, hyperthermia, mortality

## Abstract

**Purpose:**

While fever may be a presenting symptom of COVID-19, fever at hospital admission has not been identified as a predictor of mortality. However, hyperthermia during critical illness among ventilated COVID-19 patients in the ICU has not yet been studied. We sought to determine mortality predictors among ventilated COVID-19 ICU patients and we hypothesized that fever in the ICU is predictive of mortality.

**Materials and Methods:**

We conducted a retrospective cohort study of 103 ventilated COVID-19 patients admitted to the ICU between March 14 and May 27, 2020. Final follow-up was June 5, 2020. Patients discharged from the ICU or who died were included. Patients still admitted to the ICU at final follow-up were excluded.

**Results:**

103 patients were included, 40 survived and 63(61.1%) died. Deceased patients were older {66 years[IQR18] vs 62.5[IQR10], (*p* = 0.0237)}, more often male {48(68%) vs 22(55%), (*p* = 0.0247)}, had lower initial oxygen saturation {86.0%[IQR18] vs 91.5%[IQR11.5], (*p* = 0.0060)}, and had lower pH nadir than survivors {7.10[IQR0.2] vs 7.30[IQR0.2] (*p* < 0.0001)}. Patients had higher peak temperatures during ICU stay as compared to hospital presentation {103.3°F[IQR1.7] vs 100.0°F[IQR3.5], (*p* < 0.0001)}. Deceased patients had higher peak ICU temperatures than survivors {103.6°F[IQR2.0] vs 102.9°F[IQR1.4], (*p* = 0.0008)}. Increasing peak temperatures were linearly associated with mortality. Febrile patients who underwent targeted temperature management to achieve normothermia did not have different outcomes than those not actively cooled. Multivariable analysis revealed 60% and 75% higher risk of mortality with peak temperature greater than 103°F and 104°F respectively; it also confirmed hyperthermia, age, male sex, and acidosis to be predictors of mortality.

**Conclusions:**

This is one of the first studies to identify ICU hyperthermia as predictive of mortality in ventilated COVID-19 patients. Additional predictors included male sex, age, and acidosis. With COVID-19 cases increasing, identification of ICU mortality predictors is crucial to improve risk stratification, resource management, and patient outcomes.

## Introduction

Severe acute respiratory syndrome coronavirus 2 (SARS-CoV-2) was first reported in Wuhan, Hubei Province, China in December 2019. Coronavirus disease (COVID-19) has resulted in a global pandemic subsequently spreading to 188 countries and territories. Over 18.1 million people have been infected, resulting in more than 690,000 deaths world-wide,^1^ and over 4.6 million people in the United States have been infected, 154,471 of whom have died as of the manuscript submission date August 3, 2020.^2^

While overall mortality for all people infected with COVID-19 is about 0.5%,^1^ reported mortality for patients presenting to a hospital ranges widely among geographic regions, 3.4-24.3%.^3–5^ Mortality among COVID-19 patients admitted to the ICU has been variably reported from 17-88%.^5–10^ Varying ICU admission criteria, geographic locations, resource availability, and admission volume rates may contribute to disparate mortality rates across institutions and countries during the pandemic.

COVID-19 is being researched broadly and several studies have reported characteristics and outcomes of hospitalized COVID-19 patients with the intent to identify predictors and risk factors for the development of severe disease, need for ICU admission, and mortality.^3,11-15^ Described predictors for developing severe COVID-19 disease include older age, pre-existing comorbidities, obesity, hypoxia on admission, and variable biomarker abnormalities.^3,11-15^ One relevant online prediction model, the ISARIC WHO Clinical Characterisation Protocol, used a derivation dataset based on 35,463 patients from 260 hospital across England, Scotland and Wales to create the 4C Mortality Score based on 8 variables from initial hospital assessment.^16^ While this performed strongly against other prediction models, models vary in setting, predicted outcome measure, and included clinical parameters, which result in application challenges, especially when applied to small cohorts as the authors note.^16^ Less data exist regarding risk factors and predictors for mortality once a patient is already critically ill. For intensivists taking care of critically ill COVID-19 patients requiring invasive mechanical ventilation (IMV), it is crucial to identify factors which prognosticate mortality.

As our hospital surged with critically ill COVID-19 patients, we started recognizing clinical patterns of disease. One notable recurrent finding was persistent high-grade fevers in the ICU. While many studies have described fever as a presenting symptom of COVID-19 patients,^15^ few studies have identified fever as predictive of mortality.^11^ While most literature has described initial temperature upon hospital presentation to evaluate for mortality, as opposed to peak temperature during ICU admission, a recent study from affiliated hospitals in the New York area revealed body temperature on presentation ≤36°C was associated with the highest mortality and maximum temperature during hospital course correlated with mortality rate as well; they found a 42% mortality rate for body temperature >40°C.^17^ Conversely Zheng et al. suggested that fever was associated with the progression of severe COVID-19 disease^18^ and a meta-analysis of 15 articles including 2,851 COVID-19 patients found fever was not significantly associated with mortality.^19^ While conflicting research exists regarding the association of fevers with mortality in COVID-19, we anecdotally noted fevers on admission were not as high as fevers during critical illness. Additionally, patients with particularly high fevers seemed to have worse outcomes.

We therefore sought to determine predictors of mortality in critically ill COVID-19 ICU patients requiring IMV. We hypothesized that hyperthermia in the ICU is a predictor of mortality among critically ill COVID-19 patients.

## Materials and Methods

### Patient Population

This was a retrospective study of the first 108 consecutive COVID-19 positive patients admitted to the ICU requiring IMV. Patients included were admitted over a 10-week period from March 14, 2020 to May 27, 2020. The Institutional Review Board approved this study as minimal risk.

All patients requiring ICU admission and IMV, with confirmed COVID-19 by positive polymerase chain reaction (PCR) testing of nasopharyngeal sample for SARS-CoV-2, and were subsequently discharged from the ICU or who died were included. Patients who remained admitted to the ICU at the study endpoint were excluded.

Final follow-up date was June 5, 2020. At the study endpoint, 103 of 108 patients met inclusion criteria, 2 of whom remained hospitalized but were no longer in the ICU. The 5 excluded patients were still admitted to the ICU and therefore not included.

### Setting

This study was performed at a 297-bed community hospital in Central New Jersey which is part of one of the largest healthcare systems in the state. Prior to the pandemic, the ICU capacity was 16 beds, with an average ICU census of 10, and a daily average IMV census of 3. During the study period at the height of the pandemic, the ICU capacity was increased to 35 beds with additional patients boarding in the emergency department. At the peak of the crisis, 38 patients required IMV simultaneously. Board certified surgical and anesthesia critical care intensivists provided care to all ICU patients 24 hours a day, 7 days a week. Workflow and structural changes were made to provide additional surgical intensivist surge coverage.

### Data Collection and Definitions

Data were abstracted from the electronic medical record (Allscripts—Sunrise Clinical Manager, Chicago, IL). A standardized data abstraction form was created and important variables were defined. Abstractors were trained on the utilization of the data abstraction form prior to data gathering. Patient assignment was not blinded to the abstractors and the abstractors’ performance was monitored by the principal investigator. Interrater reliability and interrater agreement was not monitored. Data collected from the medical record included patient demographics, pre-existing comorbidities, vital signs, laboratory tests, imaging studies, complications, and outcomes including length of stay, ventilator days, and mortality. Temperatures were measured either by temperature sensing bladder, rectal, esophageal, or central venous probes.

High-grade fever was defined as temperature greater or equal to 103 degrees Fahrenheit (°F). Treatment using targeted temperature management systems was per the discretion of the attending intensivist and limited to availability. As previously reported, acute kidney injury (AKI) was based on the Kidney Disease: Improving Global Outcomes (KDIGO) definition as an increase in serum creatinine by 0.3 mg/dL within 48 hours or an increase of at least 1.5 times baseline within 7 days.^20^ Acute hepatic injury was defined as aspartate aminotransferase or alanine transaminase 15 times the upper limit of normal. Acute respiratory distress syndrome (ARDS) was defined by the Berlin criteria.^21^

Secondary infectious complications including pneumonia, bacteremia, and urinary tract infections were defined by positive culture data. *Clostridium difficile* infection was defined by positive stool PCR testing. Shock was defined by a vasopressor requirement to maintain a mean arterial pressure greater than 65 mmHg. New arrhythmias associated with hemodynamic instability, myocardial infarctions with increased biomarkers or new abnormalities on electrocardiography, and cardiomyopathy with newly depressed contractility on transthoracic echocardiography defined cardiac complications. Chest radiograph was used to define a new pneumothorax. Deep vein thrombosis on duplex ultrasonography or pulmonary embolism on computed tomography angiography defined venous thromboembolism. New ischemic lesions or intracranial hemorrhage on computed tomography or magnetic resonance imaging, or seizures identified by electroencephalogram defined neurologic complications.

### Statistical Analysis

Median and interquartile range (IQR) were reported for all continuous variables, since most were determined to be non-normally distributed according to the Shapiro-Wilk statistic.^22^ The significance of bivariate associations were tested using Pearson’s Chi-Square test, except when small cell counts warranted use of Fisher’s Exact test; continuous variables were compared using 2-sided Wilcoxon Rank-Sum test. For variables found to be significantly associated in bivariate analysis (*p* < 0.05), risk ratios (RR) were estimated using modified (i.e., robust variance estimator) Poisson regression.^23^ For ease of interpretation, continuous predictors were modeled as binary based on clinical cut points (88% oxygen saturation, creatinine >1.2 mg/dL, lactate >2 mmol/L, ferritin >335 ng/mL, pH nadir < 7.2, peak temperature ≥104°F). Multivariable regression analyses subsequently estimated the risk of mortality for patients with peak temperature of at least 104.0°F (vs. lower) adjusted for age and sex (Model 1); age, sex, and pH nadir (Model 2); and age, sex, pH nadir, initial oxygen saturation, and lowest P/F ratio (Model 3).

## Results

### Overall Patient Characteristics and Outcomes

Between March 14, 2020 and May 27, 2020, 180 ICU patients were tested for COVID-19 and 128 (71.1%) were COVID-19 positive, of those 108 (84.4%) required IMV. At the final date of follow-up, 103 COVID-19 positive ICU patients who required IMV met study inclusion criteria.

The median patient age was 64 years (range 25-89; IQR [15]), with 21 patients (20.4%) who were 75 years or older (Table 1). 70 were males (68%) and the majority of patients were Caucasian (46 [44.7%]), with the next largest ethnicity being Hispanic patients (34 [33%]). Hypertension was the most common comorbid condition (65 [63.1%]), followed by obesity with a body mass index >30 kg/m^2^ (56 [54.4%]), and diabetes (45 [43.7%]). Nearly half of the studied population (50 patients [48.5%]) presented to a health care provider prior to hospitalization and 40 patients (38.8%) had known high risk exposure to COVID-19.

**Table 1.** Baseline Characteristics of Critically Ill Mechanically Ventilated Patients With COVID-19.

	All Ventilated COVID-19 ICU Patients (n = 103)	Survived COVID-19 ICU Patients (n = 40)	Deceased COVID-19 ICU Patients (n = 63)	p-value^c^
Age, median (IQR), years	64.0 (15.0)	62.5 (10.0)	66.0 (18.0)	0.0237^b^
Sex, male (n, %)	70 (68.0)	22 (55.0)	48 (76.2)	0.0247
Race/Ethnicity				
Caucasian	46 (44.7)	18 (45.0)	28 (44.4)	0.4010^c^
Black	16 (15.5)	9 (22.5)	7 (11.1)	
Hispanic	34 (33.0)	11 (27.5)	23 (36.5)	
Asian	7 (6.8)	2 (5.0)	5 (7.9)	
Comorbities				
None	14 (13.6)	4 (10.0)	10 (15.9)	0.3966
Chronic Respiratory Disease				
Chronic Obstructive Pulmonary Disease/Asthma	11 (10.7)	4 (10.0)	7 (11.1)	>0.999^c^
Obstructive Sleep Apnea	9 (8.7)	6 (15.0)	3 (4.7)	0.0867^c^
Diabetes	45 (43.7)	19 (47.5)	26 (41.3)	0.5344
Obesity				
Body Mass Index >30 kg/m^2^	56 (54.4)	23 (59.0)	33 (55.9)	0.7658
Body Mass Index >35 kg/m^2^	28 (27.2)	10 (25.6)	18 (30.5)	0.6016
Cardiovascular Disease				
Hypertension	65 (63.1)	25 (62.5)	40 (63.5)	0.9190
Heart Failure	14 (13.6)	6 (15.0)	8 (12.7)	0.7397
Coronary Artery Disease	18 (17.5)	5 (12.5)	13 (20.6)	0.2893
Myocardial Infarction	6 (5.8)	0 (0.0)	6 (9.5)	0.0792^c^
Chronic Kidney Disease	12 (11.7)	6 (15.0)	6 (9.5)	0.5305^c^
End Stage Renal Disease requiring Dialysis	2 (1.9)	1 (2.5)	1 (1.6)	>0.999^c^
Cirrhosis	2 (1.9)	1 (2.5)	1 (1.6)	>0.999^c^
Immunocompromised	6 (5.8)	1 (2.5)	5 (7.9)	0.4007^c^
Rheumatologic Disease	5 (4.9)	2 (5.0)	3 (4.8)	>0.999^c^
Cognitive Disability	14 (13.6)	6 (15.0)	8 (12.7)	0.7397

Abbreviations: IQR, interquartile range; ICU, intensive care unit.

^a^ Except where indicated otherwise, bivariate comparisons tested by Pearson Chi-Square Test ^b^Bivariate comparison tested by 2-sided Wilcoxon Rank-Sum Test ^c^Bivariate comparison tested by 2-sided Fisher’s Exact Test due to small cell counts.

Initial vital signs and diagnostic study results are summarized in Table 2. Peak temperatures during critical illness in the ICU were significantly higher than admission temperatures for COVID-19 patients requiring IMV {103.3°F [IQR 1.7] vs 100.0°F [IQR 3.5] (*p* < 0.0001)}. The median difference between admission and peak temperatures was 3.2°F [IQR 2.6, range 0-10.7°F]. The initial oxygen saturation in the emergency department was 88% [IQR 16]. Most patients were hemodynamically sufficient upon presentation.

**Table 2.** Vital Signs and Laboratory Results of Critically Ill Patients With COVID-19.

	All Ventilated COVID-19 ICU Patients (N = 103)	Survived COVID-19 ICU Patients (n = 40)	Deceased COVID-19 ICU Patients (n = 63)	p-value^a^
Admission Vital Signs, median (IQR)				
Temperature, degrees Fahrenheit	100.0 (3.5)	99.8 (2.6)	100.1 (3.6)	0.5604
Heart Rate, beats per minute	99.0 (28.0)	100.5 (25.0)	98.0 (33.0)	0.7478
Systolic Blood Pressure, mmHg	132.0 (32.0)	135.5 (44.5)	130.0 (30.0)	0.3662
Mean Arterial Pressure	93.0 (23.0)	94.0 (30.0)	93.0 (22.0)	0.7697
Initial O2 Saturation	88.0 (16.0)	91.5 (11.5)	86.0 (18.0)	0.0060
Admission Laboratory Results				
White Blood Cell Count, x10^9^/L	7.7 (6.1)	7.9 (4.8)	7.6 (6.6)	0.6972
Absolute Lymphocyte Count, x10^9^/L	6.0 (7.2)	6.9 (8.0)	5.8 (7.2)	0.2819
Sodium, mmol/L	135.0 (6.0)	135 (5.5)	135 (6.0)	0.3351
Creatinine, mg/dL	1.1 (0.8)	0.9 (0.6)	1.2 (1.0)	0.0286
Total Bilirubin, mg/dL	0.5 (0.3)	0.5 (0.3)	0.5 (0.3)	0.8120
Alkaline Phosphatase, iu/L	79.0 (42.0)	82.0 (39.0)	78.0 (48.0)	0.4587
Aspartate Aminotransferase, units/L	51.0 (40.0)	46.5 (51.0)	55.0 (38.0)	0.6872
Lactate, mmol/L	1.8 (1.4)	1.7 (1.0)	2.0 (1.7)	0.0214
Prothrombin Time, sec	10.9 (1.5)	10.6 (1.8)	11.0 (1.4)	0.0722
Admission Studies				
Bilateral Infiltrates on Chest X-ray, n (%)	94 (91.3%)	36 (90.0%)	58 (92.1%)	0.7325^c^
Chest CT scan Obtained, n (%)	22 (21.4%)	10 (25.0%)	12 (20.0%)	0.5543^b^
False Negative COVID-19 Tests, n (%)	5 (4.9%)	3 (7.7%)	2 (3.2%)	0.3678^c^
Highest Value During Hospitalization				
Lactate Dehydrogenase, U/L	566.0 (328.0)	527.0 (293.0)	571.0 (349.0)	0.2040
Ferritin, ng/mL	1161 (1376.2)	1020 (935.0)	1334 (1832.5)	0.0197
Triglycerides, mg/dL	197.0 (188.0)	182.0 (175.0)	200.0 (187.0)	0.6016
D-Dimer, mg/L	5.2 (12.7)	5.8 (9.5)	4.5 (20.3)	0.6741
Fibrinogen, mg/dL	634.0 (288.0)	673.0 (269.7)	613.0 (271.0)	0.3568
pH Nadir	7.20 (0.2)	7.30 (0.2)	7.10 (0.2)	<0.0001
Lowest P/F Ratio	72.0 (48.8)	94.2 (71.8)	60.5 (36.9)	<0.0001
Temperature Peak (degrees Fahrenheit)	103.3 (1.7)	102.9 (1.4)	103.6 (2.0)	0.0008
High Grade Fever (≥103 degrees Fahrenheit)	58 (56.3)	18 (45)	40 (63.5)	0.0408
High Grade Fever (≥104 degrees Fahrenheit)	31 (30.1)	5 (12.5)	26 (42.6)	0.0013
High Grade Fever (≥105 degrees Fahrenheit)	14 (13.6)	0	14 (22.2)	

Abbreviations: PF, arterial oxygen partial pressure to fractional inspired oxygen.

^a^ Except where indicated otherwise, bivariate comparisons tested by 2-sided Wilcoxon Rank-Sum Test.

^b^ Bivariate comparison tested by Pearson Chi-Square test.

^c^ Bivariate comparison tested by Fisher’s Exact Test due to small cell counts.

102 of the 103 IMV patients had ARDS, and 76 (73.8%) had severe ARDS (Table 3). 74 (71.8%) developed acute kidney injury and 28 (27.2%) received renal replacement therapy. Concomitant bacterial pneumonia was diagnosed in 42 patients (40.8%) and 22 patients (21.3%) developed bacteremia.

**Table 3.** Treatments Provided, Complications, and Outcomes of COVID-19 ICU Patients.

	All COVID-19 ICU Patients (n = 103)	Survivors COVID-19 ICU Patients (n = 40)	Deceased COVID-19 ICU Patients (n = 63)	p-value^a^
Remdesivir	14 (13.6)	5 (12.5)	9 (14.3)	0.7727
Tocilizumab	40 (38.9)	15 (37.5)	25 (39.7)	0.8247
Convalescent Plasma	9 (8.7)	1 (2.5)	8 (12.7)	0.1484^b^
Chemical Neuromuscular Blockade	36 (35.0)	8 (20)	28 (44.4)	0.0140
Prone Positioning	24 (23.3)	4 (10)	20 (31.7)	0.0109
ARDS	102 (99.0)	39 (97.5)	63 (100)	0.3883^b^
Mild ARDS	6 (5.8)	3 (7.5)	3 (4.8)	0.6751^b^
Moderate ARDS	22 (21.4)	15 (37.5)	7 (11.1)	0.0014
Severe ARDS	76 (73.8)	22 (55)	54 (85.7)	0.0006
Shock Resulting in Vasopressor Requirement	91 (88.3)	30 (75)	61 (96.8)	0.0026^b^
Infectious Complications				
Bacterial Pneumonia	42 (40.8)	21 (52.5)	21 (33.3)	0.0620
Urinary Tract Infection	20 (19.4)	8 (20)	12 (19)	0.9052
Bacteremia	22 (21.3)	5 (12.5)	17 (27)	0.0805
Clostridium Difficile	2 (1.9)	1 (2.5)	1 (1.6)	>0.999^b^
Acute Kidney Injury	74 (71.8)	22 (55)	52 (82.5)	0.0041
Renal Replacement Therapy	28 (27.2)	9 (22.5)	19 (30.2)	0.3945
Acute Hepatic Injury	7 (6.8)	0	7 (11.1)	0.0411^b^
Venous Thromboembolism				
Deep Vein Thrombosis	3 (2.9)	3 (7.5)	0	0.0559^b^
Pulmonary Embolism	3 (2.9)	1 (2.5)	2 (3.2)	>0.999^b^
Cardiac Complications				
Arrhythmia	32 (31.1)	10 (25)	22 (34.9)	0.2890
Myocardial Infarction	4 (3.9)	0	4 (6.3)	0.1554^b^
Cardiomyopathy	8 (7.8)	1 (2.5)	7 (11.1)	0.1462^b^
Pneumothorax	8 (7.8)	3 (7.5)	5 (7.9)	>0.999^b^
Neurological Complications				
Seizures	2 (1.9)	1 (2.5)	1 (1.6)	>0.999^b^
Cerebrovascular Accident	2 (1.9)	1 (2.5)	1 (1.6)	>0.999^b^
Intracranial Hemorrhage	2 (1.9)	1 (2.5)	1 (1.6)	>0.999^b^
Invasive Mechanical Ventilation (IMV)				
Ventilator on admission, n (%)	36 (35.0)	11 (27.5)	25 (39.7)	0.2063
Hospital days prior to IMV, median (IQR)*	3.0 (2.0)	3.0 (2.0)	3.0 (4.0)	0.9130
** **Ventilator Days, median (IQR)	9.0 (11.0)	10.0 (11.0)	8.0 (10.0)	0.7759
ICU Length of Stay, median (IQR), days	11.0 (12.0)	14.0 (13.0)	9.0 (10.0)	0.0400
Hospital Length of Stay, median (IQR), days	16.0 (13.0)	22.0 (13.0)	12.0 (9.0)	<0.0001

Abbreviations: ICU, intensive care unit; IQR, Interquartile Range; IMV, invasive mechanical ventilation; ARDS, acute respiratory distress syndrome.

^a^ Except where indicated otherwise, bivariate comparisons tested by Pearson Chi-Square test or 2-sided Wilcoxon Rank-Sum Test (continuous variables).

^b^ Bivariate comparison tested by Fisher’s Exact Test due to small cell counts.

* Hospital days prior to IMV calculated among patients who did not require IMV upon admission.

### Characteristics and Outcomes for Deceased Patients Versus Survivors

Among the 103 ventilated COVID-19 ICU patients, 63 died (61.1%). Deceased patients were older {median age 66 years [IQR 18] vs. 62.5 years [IQR 10] (*p* = 0.0237)} and more often male {48 (68%) vs. 22 (55%) (*p* = 0.0247) (Table 1)}. There was not a significant difference regarding race and ethnicity, although 33% of all patients were Hispanic and 23 Hispanic patients died accounting for 36.5% of all deceased patients. Comorbidities among patients did not significantly differ, however all patients with a history of myocardial infarction died (6 [5.8%]).

Initial oxygen saturation was significantly lower among decedents {median 86.0 [IQR 18] vs 91.5 [IQR 11.5] (*p* = 0.0060)}. There were no significant differences among other vital signs on admission with respect to survival (Table 2). The initial laboratory results that differed by mortality status were creatinine {median 1.2 mg/dL [IQR 1.0] vs 0.9 mg/dL [IQR 0.6] (*p* = 0.0286)} and lactate {median 2.0 mmol/L [IQR 1.7] vs 1.7 mmol/L [IQR 1.0] (*p* = 0.0214)}, both of which were significantly higher among deceased. Peak ferritin levels were high overall (median 1161.0 ng/mL [IQR 1376.2]) and significantly higher among deceased patients {median 1334 ng/mL [IQR 1832.5] vs. median 1020 ng/mL [IQR 935] (*p* = 0.0197)}.

Acidosis was ubiquitous among all COVID-19 patients requiring IMV. The deceased patients were found to have a significantly lower pH nadir than survivors {7.10 [IQR 0.2] vs 7.30 [IQR 0.2] (*p* < 0.0001)} (Table 2). Deceased patients were also found to have a significantly lower P/F ratio nadir than survivors {60.5 [IQR 36.9] vs 94.2 [IQR 71.8] (*p* < 0.0001)}, which correlated with significantly more deceased patients having severe ARDS (*p* = 0.0006) and moderate ARDS (*p* = 0.0014) as compared to survivors (Table 3). Shock requiring vasopressors, acute kidney injury, and acute hepatic injury were significantly higher among deceased patients (all *p* < 0.05) (Table 3).

Deceased patients had a shorter ICU length of stay {9 days [IQR 10] vs 14 days [IQR 13] (*p* = 0.0400)} and a shorter hospital length of stay {12 days [IQR 9] vs 22 days [IQR 13] (*p* < 0.0001)} as compared to survivors (Table 3). There was no difference in ventilator days or the need for emergent intubation on admission among survivors compared to deceased patients.

### Hyperthermia as Related to Mortality

**Figure 1.**
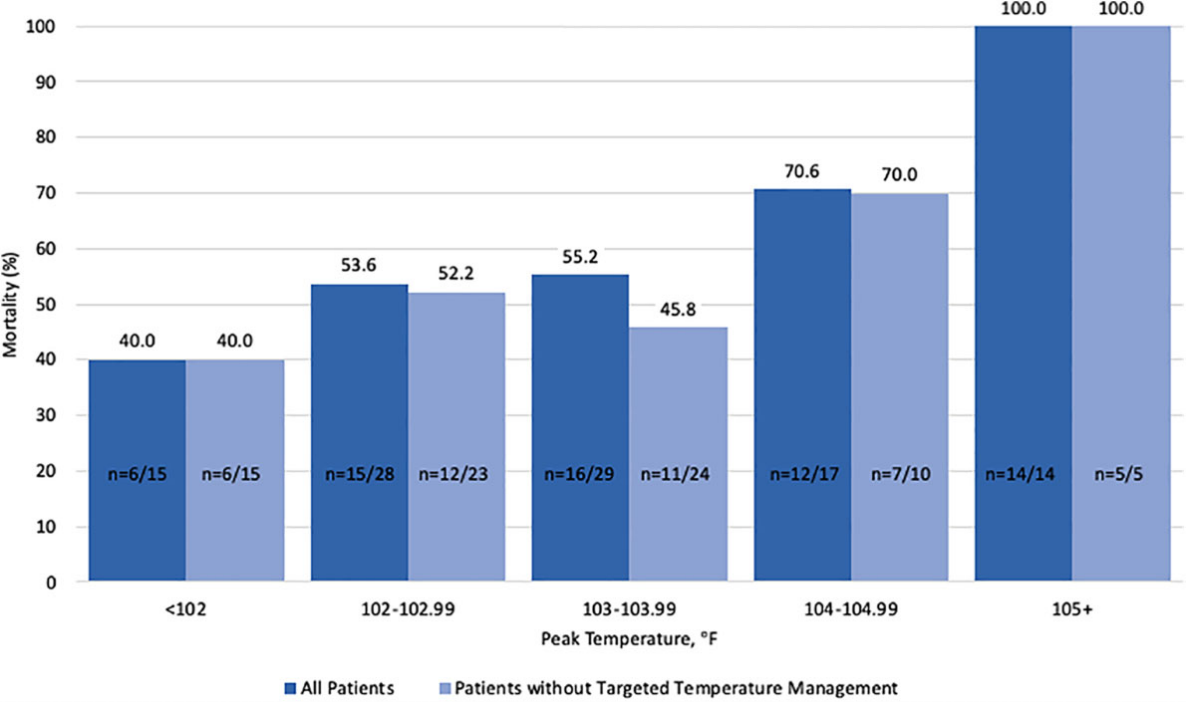
A direct relationship between temperature and mortality: as peak temperature increased, mortality increased as well among COVID-19 mechanically ventilated patients.

Although there was no difference in admission temperature among deceased patients compared to survivors (100.1 [IQR 3.6] vs 99.8°F [IQR 2.6]), peak temperature in the ICU was significantly higher among decedents {median 103.6°F [IQR 2.0] vs 102.9°F [IQR 1.4] (*p* = 0.0008) (Table 2)}. High grade fevers greater than 103°F during critical illness in the ICU were significantly more common among the deceased patients. A direct relationship was identified between temperature and mortality: as peak temperature increased among COVID-19 IMV patients, mortality increased as well (Figure 1). While overall mortality was 61.1% for the studied population, mortality was lower (40%) among patients with peak temperature less than 102°F. Conversely, mortality was higher (70.6%) among patients with peak temperature greater than 104°F and there was 100% mortality among the 14 patients who had hyperthermia greater than 105°F.

There was no difference in outcome among the 11 patients treated with a non-invasive targeted temperature management system (Arctic Sun^TM^ Temperature Management System, Covington, GA). Conversely of the 18 patients treated with invasive temperature management systems (ZOLL, ZOLL Medical Corporation), 2 survived. Overall 26 (25.2%) COVID-19 ICU patients received targeted temperature management (3 patients received both non-invasive and invasive temperature management systems during their hospital course), among that population 21 (80.8%) died and 5 (19.2%) survived (*p* = 0.018).

### Predictors of Mortality

In univariable analysis, risk of death increased by about 1% with each year of age (RR, 1.01; 95% confidence interval [CI], 1.00-1.03) and was 51% higher among males than females (RR 1.51; 95% CI, 1.01-2.26) (Table 4A). Having an initial oxygen saturation less than 88% was associated with 59% (RR, 1.59; 95% CI, 1.14-2.21) increased risk of death, and patients with pH nadir less than 7.2 had more than 2.5 times (RR 2.58; 95% CI, 1.65-4.05) risk of death relative to those with higher pH. Mortality risk for those with peak temperature of at least 103°F was 41% higher (RR, 1.41; 95% CI, 0.99-2.01) than those with a lower peak temperature and 68% higher (RR, 1.68; 95% CI, 1.27-2.22) if peak temperature was greater than 104°F.

**Table 4A.** Univariable Analysis of Mortality Risk Factors for Critically Ill Patients With COVID-19 (n = 103).

	β	RR (95% CI)	p value
Demographics			
Age, years	0.0136	1.01 (1.00, 1.03)	0.0501
Sex, Male	0.4112	1.51 (1.01, 2.26)	0.0472
Admission Vitals and Labs			
Initial O_2_ Saturation, < 88	0.4637	1.59 (1.14, 2.21)	0.0056
Creatinine, >1.2 mg/dL	0.2545	1.29 (0.96, 1.74)	0.0960
Lactate, >2 mmol/L	0.3049	1.36 (1.00, 1.84)	0.0514
Hospitalization Labs			
Peak Ferritin, >335 ng/mL	0.6643	1.94 (0.76, 4.96)	0.1645
pH Nadir, < 7.2	0.9484	2.58 (1.65, 4.05)	<0.0001
Temperature Peak, ≥ 103 degrees Fahrenheit	0.3451	1.41 (0.99, 2.01)	0.0542
Temperature Peak, ≥ 104 degrees Fahrenheit	0.5173	1.68 (1.27, 2.22)	0.0003
Lowest P/F Ratio	-0.0084	0.99 (0.99, 1.00)	0.0041

The effect of peak temperature, as well as age, sex, and pH nadir, remained significant in multivariable analysis as predictors of mortality. Whereas, initial oxygen saturation and lowest P/F ratio were no longer significant predictors of mortality risk after adjusting for these factors (Table 4B). Peak temperature remained a significant predictor of mortality after adjusting for initial oxygen saturation <88% and lowest P/F ratio. When adjusting for these risk factors, mortality was 2% higher with age and 48% higher for men. Peak temperature was found to have an even greater impact on mortality. Mortality was 60% higher among patients with peak temperatures of 104°F than those with lower temperatures when adjusting for age sex, pH nadir, initial O2 saturation <88%, and P/F ratio nadir; this risk was even higher when adjusting for only age and sex, resulting in 75% higher mortality (aRR 1.75; 95% CI, 1.31-2.32) (Table 4B).

**Table 4B.** Multivariable Analysis of Mortality Risk Factors for Critically Ill Patients With COVID-19 (n = 103).

	Model 1	Model 2	Model 3
	aRR (95% CI)	aRR (95% CI)	aRR (95% CI)
Temperature Peak, ≥ 104 degrees Fahrenheit	1.75 (1.31, 2.32)	1.67 (1.27, 2.20)	1.60 (1.20, 2.13)
Age, years	1.02 (1.00, 1.03)	1.02 (1.01, 1.03)	1.02 (1.01, 1.04)
Sex, Male	1.48 (1.02, 2.15)	1.38 (0.98, 1.96)	1.34 (0.94, 1.89)
pH Nadir, <7.2	---	2.46 (1.64, 3.69)	2.06 (1.34, 3.16)
Initial O_2_ Saturation, <88	---	---	1.27 (0.94, 1.72)
Lowest P/F Ratio	---	---	1.00 (0.99, 1.00)

Abbreviations: RR, Risk Ratio; aRR, Adjusted Risk Ratio; CI, Confidence Interval.

^a^ Risk ratios estimated using modified (robust estimator) Poisson regression.

Univariate analysis was repeated among the 77 critically ill COVID-19 patients who did not receive targeted temperature management (Table 5A), results were similar. Mortality risk for those with peak temperature of at least 103°F was 68% higher (RR, 1.68; 95% CI, 1.17-2.43) than those with a lower peak temperature and 97% higher (RR, 1.97; 95% CI, 1.57-2.48) if peak temperature was greater than 104°F. Multivariable analysis was also repeated among the 77 patients who did not receive targeted temperature management (Table 5B). Mortality was 66% higher among patients with peak temperatures of 104°F than those with lower temperatures when adjusting for age and sex; this result was even higher when adjusting for age, sex, pH nadir, initial O2 saturation <88%, and P/F ratio nadir, resulting in 87% higher mortality (aRR 1.87; 95% CI, 1.27-2.75).

**Table 5A.** Univariable Analysis of Mortality Risk Factors for Critically Ill Patients With COVID-19 Who Did Not Receive Targeted Temperature Management (n = 77).

	β	RR (95% CI)	p value
Demographics			
Age, years	0.0379	1.04 (1.02, 1.06)	<0.0001
Sex, Male	0.4765	1.61 (0.97, 2.68)	0.0657
Admission Vitals and Labs			
Initial O_2_ Saturation, <88	0.4215	1.52 (1.01, 2.29)	0.0424
Creatinine, >1.2 mg/dL	0.3947	1.48 (1.00, 2.21)	0.0520
Lactate, >2 mmol/L	0.4164	1.52 (1.01, 2.28)	0.0446
Hospitalization Labs			
Peak Ferritin, >335 ng/mL	0.7267	2.07 (0.63, 6.79)	0.2307
pH Nadir, <7.2	1.10	3.01 (1.78, 5.09)	<0.0001
Temperature Peak, ≥103 degrees Fahrenheit	0.5204	1.68 (1.17, 2.43)	0.0052
Temperature Peak, ≥104 degrees Fahrenheit	0.6792	1.97 (1.57, 2.48)	<0.0001
Lowest P/F Ratio	-0.0070	0.99 (0.99, 1.00)	0.0292

**Table 5B.** Multivariable Analysis of Mortality Risk Factors for Critically Ill Patients With COVID-19 Who Did Not Receive Targeted Temperature Management (n = 77).

	Model 1	Model 2	Model 3
	aRR (95% CI)	aRR (95% CI)	aRR (95% CI)
Temperature Peak, ≥ 104 degrees Fahrenheit	1.66 (1.09, 2.51)	1.88 (1.33, 2.68)	1.87 (1.27, 2.75)
Age, years	1.04 (1.02, 1.06)	1.04 (1.02, 1.05)	1.04 (1.02, 1.05)
Sex, Male	1.70 (1.10, 2.62)	1.59 (1.07, 2.37)	1.62 (1.06, 2.46)
pH Nadir, <7.2	---	2.73 (1.71, 4.35)	2.47 (1.49, 4.07)
Initial O_2_ Saturation, <88	---	---	1.27 (0.88, 1.84)
Lowest P/F Ratio	---	---	1.00 (0.99, 1.00)

Abbreviations: RR, Risk Ratio; aRR, Adjusted Risk Ratio; CI, Confidence Interval a Risk ratios estimated using modified (robust estimator) Poisson regression.

## Discussion

This retrospective cohort study identified fever, male sex, increasing age, and acidosis as predictive of mortality in ventilated COVID-19 patients. Prediction models for developing severe COVID-19 infections requiring ICU admission are emerging within the academic literature^3,15^; however there are limited data regarding predictors of mortality among patients who already have severe disease. This work represents one of the first studies to identify hyperthermia in the ICU as a predictor of mortality in mechanically ventilated COVID-19 patients.

As the COVID-19 pandemic spread, fever was identified as a common presenting symptom and predictor of the presence of COVID-19.^15^ However, prior studies that sought to determine predictors of mortality in hospitalized COVID-19 patients did not identify fever as a risk factor. ^3,11-15,19^ These studies typically used temperature upon hospital presentation for the evaluation. In our cohort, median temperature upon hospital presentation was consistent with a low-grade fever (100.0°F [IQR 3.5]) and not significantly different among survivors compared to decedents; accordingly, temperature on admission was not predictive of mortality. Conversely, peak temperature during critical illness among ventilated COVID-19 patients in the ICU was significantly higher than admission temperature and consistent with high-grade fever (103.3°F [IQR 1.7] vs 100.0°F [IQR 3.5]) (*p* < 0.0001).

Moreover, high-grade fevers were found to be common among COVID-19 ICU patients, with 56.3% of patients experiencing peak temperatures of 103°F and 30.1% greater than 104°F. When evaluating peak temperature in the ICU, it was significantly higher among deceased COVID-19 patients as compared to survivors (*p* = 0.0008). Furthermore, multivariate analysis confirmed hyperthermia in the ICU to be a significant predictor of mortality and resulted in a 75% greater risk of mortality (Table 4). This study went on to demonstrate a direct correlation between temperature and mortality: as peak temperature increased among COVID-19 IMV patients, mortality increased as well, with 100% mortality in the 14 patients with fevers greater than 105°F.

The purpose in identifying predictors of mortality is not only to develop prognostic information but also to identify predictors that expedite intervention to improve outcomes. While predictors such as age and gender cannot be changed, hyperthermia can be treated and normothermia can be achieved; however, it is unclear whether targeted temperature management is beneficial or harmful in the setting of sepsis. The CASS study, a randomized controlled trial of 436 patients with severe sepsis or septic shock were randomized to routine thermal management or induced hypothermia (target temperature 32-34ºC) for 24 hours followed by normothermia (36-38ºC) for 48 hours; the trial was stopped for futility as 44.2% of patients in the hypothermia group died compared to 35.8% in the routine thermal management group (*p* = 0.07).^24^ A second randomized controlled trial in patients who presented to the emergency department with septic shock were treated with standard fever control vs intensive fever control maintaining normothermia below 37ºC; this study was stopped secondary to a slow enrollment rate and the presence of adverse effects, namely shivering, in the therapeutic normothermia group.^25^

While there has been some controversy as to whether normothermia should be pursued in febrile ICU patients as it could be harmful, evidence from Schortgen et al demonstrated fever control using external cooling devices in a randomized controlled trial reduced 14-day mortality in ICU patients with septic shock; however this study had limitations including lack of power to examine mortality, baseline differences among the 2 groups with the control group being more critically ill, and ultimately no difference in mortality at ICU or hospital discharge.^26^ While, mortality benefits of fever control in septic shock include decreased oxygen consumption, hemodynamic stabilization, and decreased vasopressor requirements, meta-analyses of randomized controlled trials did not support that more active fever management increased survival compared to less active fever management in patients with limited physiological reserves.^27,28^ Retrospective and observational studies have even suggested benefit to elevated temperatures which in turn enhance the immune system and inhibit pathogen infectivity.^29–35^

Our practice pattern was to utilize external or systemic temperature management devices to combat severe hyperthermia with the goal of inducing normothermia, not hypothermia. This practice pattern was left to the discretion of the attending intensivist as temperature management devices were limited and continually triaged among patients with the most severe hyperthermia. While it cannot be determined whether temperature management had a direct effect on outcomes as the devices were triaged to the most critically ill patients, the majority of patients who died {40 patients (63.5%)}, did not receive targeted temperature management but still experienced high grade fevers; this supports the findings in this study that high grade fevers were common and associated with increased mortality. Additionally, when repeating univariable and multivariable analyses of the 77 patients who did not receive targeted temperature management, the results were similar indicating mortality remained higher among patients with higher peak temperatures; this suggests while targeted temperature management could be a confounder in this study, when it was removed from analysis, peak hyperthermia remained significantly associated with mortality.

Our study has important limitations. This was a retrospective observational trial without intervention performed for analysis. While continuous predictors were modeled as binary based on clinical cut points for ease of interpretation in the multivariable analysis, this could potentially uncover nonlinear relationships or inflate the type I error rate. A confounder within this study is the non-uniform use of temperature management systems and the inability to determine the effect on outcomes. Future studies are therefore needed to determine whether targeted temperature management in COVID-19 ICU patients results in a mortality benefit. Currently a pilot randomized controlled trial (ClinicalTrials.gov Identifier: NCT02706275) is enrolling septic patients, including COVID-19 patients, comparing outcomes in patients externally warmed to 1.5ºC greater than their baseline minimum temperature within the previous 24 hours to a control group that receives standard body temperature management. As this pandemic continues and ICU COVID-19 volumes continue to surge, it is important to be aware of the effect of hyperthermia on mortality and to determine the optimal temperature management strategy to improve patient outcomes.

## Conclusion

In this retrospective study of critically ill mechanically ventilated COVID-19 patients admitted to a community hospital ICU, predictors of mortality were ICU hyperthermia, male sex, increasing age, and acidosis. Severe hyperthermia was common and increasing temperatures were independently associated with increasing mortality rates. The utilization of targeted temperature management did not have an effect on outcomes. As COVID-19 becomes even more prevalent, identification of predictors of mortality in ICU patients is critical.
